# Influence of Cr Content on the High-Temperature Oxidation Behavior and Mechanism of Low-Alloy Steels

**DOI:** 10.3390/ma16144964

**Published:** 2023-07-12

**Authors:** Gi-Hoon Kwon, Hyunjun Park, Byoungho Choi, Young-Kook Lee, Kyoungil Moon

**Affiliations:** 1 Heat & Surface Technology R&D Group, Korea Institute of Industrial Technology, Siheung 15014, Republic of Korea; kgh9900a@kitech.re.kr (G.-H.K.); jun79s@kitech.re.kr (H.P.); byoungho@kitech.re.kr (B.C.); 2 Department of Materials Science and Engineering, Yonsei University, Seoul 03722, Republic of Korea; yklee@yonsei.ac.kr

**Keywords:** high-temperature oxidation, mass gain, chromite, oxidation rate constant, activation energy

## Abstract

The high-temperature oxidation behavior of low-carbon steel (AISI 1015, AISI 8617, AISI 4115) was investigated over the temperature range from 600 to 1000 °C in humid air containing 25% water vapor. Mass gain of oxidation measurement was performed to study the oxidation kinetics. The microstructure, thickness, and composition of the oxide scale formed were investigated via optical microscope (OM), scanning electron microscope (SEM) equipped with an energy dispersive spectrometer (EDS), X-ray diffraction (XRD), and electron probe microanalyzer (EPMA). The oxidation process was performed from 2 to 100 min. As the oxidation time increased, the trend of mass gain per unit area switched from a linear to a parabolic law, regardless of the steel grade used. As the chromium content increased, the duration of time during which the oxidation rate followed a linear relationship decreased. In the low-alloy steel with higher chromium content, the thickness of the mixed oxide layer containing Cr increased and the oxidation rate decreased at all oxidation temperatures.

## 1. Introduction

High-temperature oxidation is a process in which the surface of a metal undergoes oxidation when it is exposed to an oxidizing environment at high temperatures. This can occur when metals are in contact with gases such as oxygen, water vapor, or sulfur dioxide or when they are in contact with molten salts or other liquids that contain oxidizing species [1,2,3,4]. During high-temperature oxidation, the metal reacts with the oxidizing species to form metal oxides, which can build up on the surface of the metal and ultimately lead to degradation or failure of the material. This can have significant consequences in a variety of applications, including high-temperature industrial processes, power generation, and aerospace engineering. Microscopic cross-sections of solid-state oxide scales grown from iron surfaces under isothermal conditions (across a temperature range of 973 K to 1523 K) were first observed by Païdassi in the 1950s [5,6].

**Figure 1:**
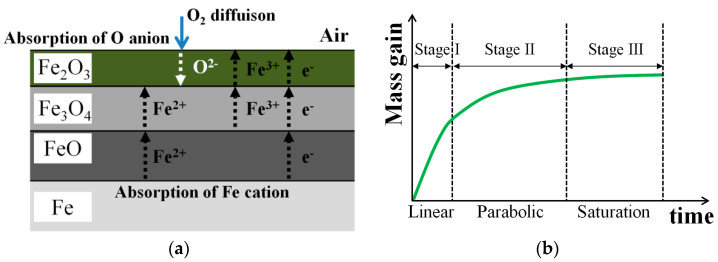
Schematic illustration of high-temperature oxidation: (**a**) mechanism of oxide formation and (**b**) three stages of oxidation reaction according to exposure time.

The formation structure of the iron oxide layer produced by the high-temperature oxidation reaction is shown in Figure 1a. First, oxygen molecules existing in the air diffuse and move around the bulk surface. When oxygen molecules are adsorbed on the surface interface, they are decomposed into the ionic form from the lower oxide scale existing on the surface. Because the diffusion of oxygen in the gas phase from the iron surface dominates over the kinetic reactions in the solid phase, hematite is formed on the outermost surface. Hematite is the most oxygen-rich, and is used for smooth outward diffusion and inward diffusion of O anions and Fe cations. As a result, an oxide film is formed in the order of magnetite and wüstite [7,8]. Figure 1b shows the mass gain change step according to the oxidation time. Mass gain change is divided into a linear step, parabolic step, and saturation step according to oxidation time [9,10,11,12]. During the initial oxidation step, the mass gain over time increases according to a linear law due to sufficient inward diffusion of oxygen until the oxide film completely covers the outermost surface of the iron [13]. This reaction can be expressed by Equation (1):

(1) $$∆W=K_{l}t$$

where Δ*W* is the oxidation weight gain per unit area (mg/mm^2^), *K_l_* is a linear reaction rate constant (mg·mm^−2^·min^−1^), and *t* is the oxidation time (min). After the linear step, the oxide film covering the iron surface due to continuous contact with oxygen hinders the movement of oxygen anions, and the oxidation rate starts to decrease until it reaches the parabolic step [7,14]. The parabolic reaction can be expressed by the formula of Kofstad [10], as shown below (Equation (2)):

(2) $${\left(∆W\right)}^{2}=K_{p}t$$

Here, *K_p_* is the parabolic rate constant (mg^2^·mm^4^·s^−1^) and *t* is the oxidation time (s). Finally, as the outward diffusion of Fe cations is blocked by the inner oxide near the parent material, Cr is added to the iron-based alloy in order to suppress this high-temperature oxidation reaction of iron. In this step, the oxidation behavior varies depending on the Cr content [15,16]. The oxidation behavior of Cr-alloyed steel easily reaches the parabolic step, as Cr oxide is formed on the surface and Fe ions and Cr elements combine with the FeO phase to form FeCr_2_O_4_ as the inner oxide [17]. Although chromite hinders the oxidation rate by forming an inner oxide, its effect becomes limited as Fe continues to diffuse outward over time [18]. Previous studies have conducted experiments on the oxidation behavior of iron according to various Cr contents. In order to improve high-temperature oxidation resistance in petrochemical processing and metal dusting environments, 20 wt.% chromium can be added, and silicon has additionally been included [19]. A small amount of Si improves the heat resistance of alloy steel and plays a role in regenerating Cr_2_O_3_, which is a passive film formed on the outmost surface [20,21].

Fe-Cr alloys with a Cr content of 8 wt.% or more form Cr_2_O_3_ on the outermost surface during the initial stage of oxidation, which hinders the availability of oxygen from inward diffusion and shortens the linear step. Because the complete deposition of Cr_2_O_3_ on the surface prevents both inward and outward diffusion of iron ions, the oxidation resistance can be greatly increased, resulting in a marked reduction in the oxidation rate [22,23]. When the Cr content in the alloy steel is lower than a specific concentration, it is difficult to form a Cr_2_O_3_ film, and there may be a rapid oxidation reaction. While there are many studies examining the high-temperature oxidation behavior of alloy steels with a Cr content of more than 5 wt.%, there are few studies on high-temperature oxidation of low-alloy steels with a Cr content of 1 wt.% or less. Mingxin Hao et.al [9] conducted temperature-dependent oxidation experiments on Fe-1Cr-0.2Si steel and Fe-0Cr-0.2Si steel to closely examine how the Cr content of the base material affects the oxidation behavior. However, from the point of view of the oxidation difference according to Cr content, it was judged that the steel type they used was insufficient resulting in the change of the oxide layer according to the oxidation time not being optically shown. Therefore, in order to observe the oxidation behavior according to the oxidation time by temperature during the high-temperature oxidation process of various steel types (AISI 1015, AISI 8617, and AISI 4115) according to the Cr content, in this study we obtained the mass gain by oxidation, the structure of the oxidation layer, and the oxidation kinetics.

## 2. Materials and Methods

The study of the high-temperature oxidation behavior of steel grades according to Cr content is carried out through tube furnace oxidation. The chemical composition of the test steel is shown in Table 1. To study the effect of Cr element on the oxidation kinetics of the tested steel, AISI 1015 and AISI 8617 steel were introduced for comparison. Each steel grade differed only in its chromium content, with the content of other elements being the same as that of AISI 4115 steel.

The experimental steels were cut into 30 mm × 10 mm by electric discharge wire cutting, then 200#, 600#, 800#, 1000#, 1500#, and 2000# SiC sandpapers were used to grind the samples until the surface of the test pieces were flat. The test pieces were cleaned in absolute ethyl alcohol using an ultrasonic cleaner and subsequently weighed using an electronic balance (A&D, Tokyo, Japan, GR-200) with a sensitivity of 0.1 mg.

**Figure 2:**
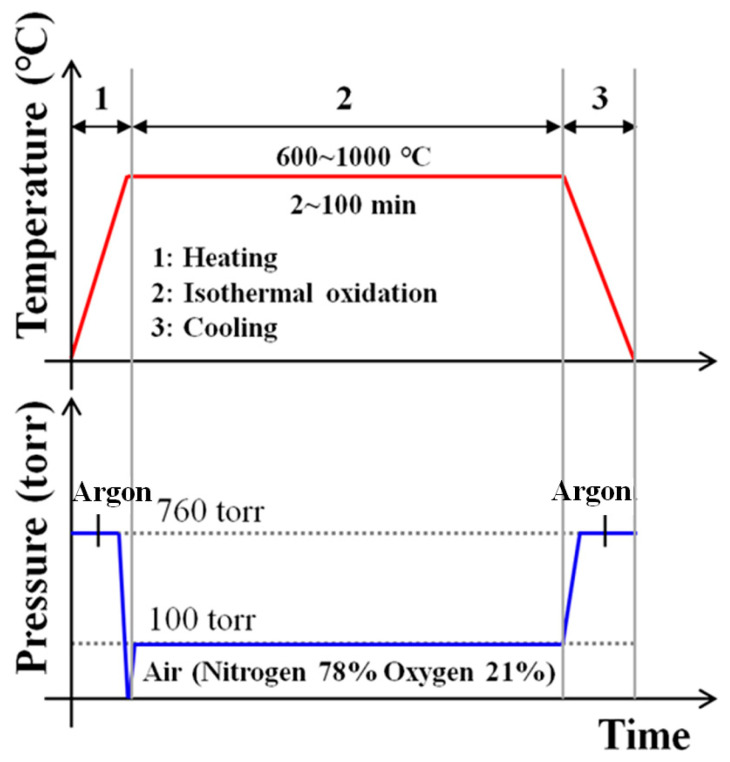
Schematic diagram of high-temperature oxidation process.

The cleaned specimens were charged in a quartz tube furnace and oxidation experiment was conducted in the order shown in Figure 2. The tube furnace was heated from room temperature to the required oxidation temperature at a rate of 14.5 °C/min, then the experimental sample was placed in the center of the tube furnace. The isothermal temperatures required for the oxidation process were 600 °C, 700 °C, 800 °C, 900 °C, and 1000 °C. After reaching the set oxidation temperature, air was introduced into the furnace at a flow rate of 50 sccm until the oxidation time (2, 5, 10, 20, 50, 100 min) was finished. At the end of the oxidation step, the heater was turned off and cooled to room temperature at a rate of 7.2 °C/min by injecting argon gas, then weighed again at different oxidation times to determine the mass gains. The phase analysis of the scale was observed by metallographic microscopy, energy-dispersive spectroscopy, and X-ray diffraction, and electron probe microanalysis. To evaluate the corrosion resistance after the oxidation test, the weight changes of the specimens before and after oxidation were measured to 0.0001 g. After polishing the cross-section of the oxidized specimens using SiC abrasive paper and 1 μm diamond suspension, the microstructures of the oxidized specimens were observed using an optical microscope (Huvitz, Anyang-si, Republic of Korea, HRM-300) and a scanning electron microscope (FEI Hong Kong Company, Hong Kong, China, NNS-450). In addition, the surfaces of the oxide specimens were analyzed using an X-ray diffractometer (Rigaku, Tokyo, Japan, Miniflex II); the Cu K_α_ target was used, the diffraction angle was 20 to 90°, and the current and voltage were 30 mA and 40 kV, respectively. An electron probe microanalyzer (JEOL, Tokyo, Japan, JXA-8500F) was used to measure the oxygen concentration gradient in the direction of oxidation depth, with the current, voltage, and electron beam focus size set to 20 nA, 15 kV, and 1 μm^2^, respectively. In order to distinguish the hardness of each phase of the oxide layer, nanoindentation tests were carried out using a nanoindenter (Helmut Fischer, Sindelfingen, Germany, HM-2000). The tip of this nanoindenter is a 4 side Vickers diamond pyramid indenter. The load and indentation time were set to 2 mN and 20 s, respectively, and the measurement was repeated five times. 

## 3. Results and Discussion

**Figure 3:**
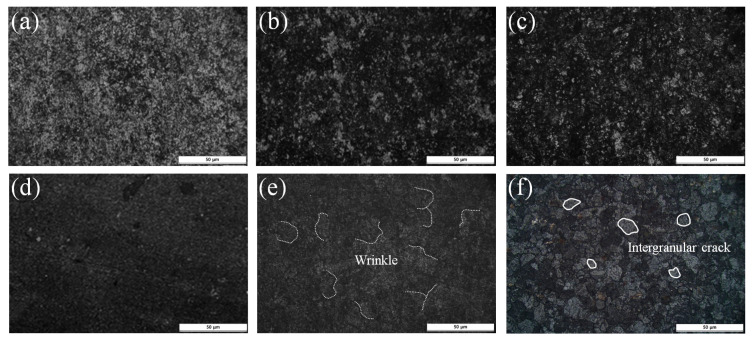
Top views of AISI 4115 specimens oxidized at 1000 °C according to exposure time in the furnace: (**a**) 2 min; (**b**) 5 min; (**c**) 10 min; (**d**) 20 min; (**e**) 50 min; (**f**) 100 min.

Figure 3 shows the microscopic morphology of the oxide scale obtained by high-oxidation testing. It can be seen that the surface shows a distinct difference after isothermal oxidation according to exposure time. The surface color of the oxidized specimen exposed for 10 min is almost gray–black, and the color tends to become darker as the exposure time increases. In particular, the surface of the specimen oxidized for more than 50 min is black and partially contains oxidation wrinkles with a length of about 30 μm. Finally, intergranular cracks can be observed on the surface of the specimen oxidized for 100 min, and the cracks have caused spalling. This is due to thermal stress generated by the difference in the thermal expansion coefficient between the oxide film and defects such as pores that exist in the oxide film under a high-temperature environment [24]. 

**Figure 4:**
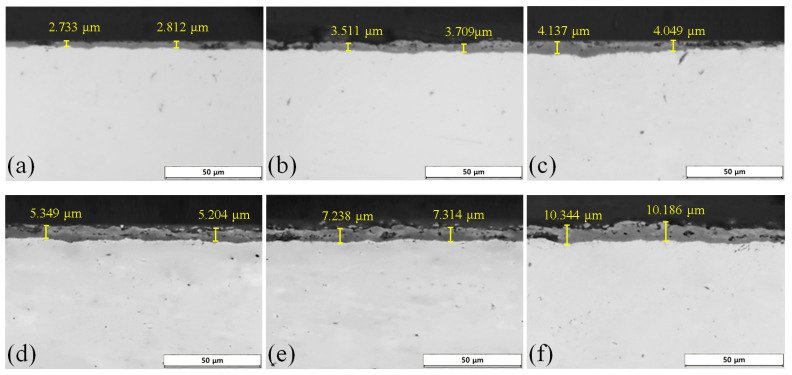
Optical cross-section metallographic pictures of AISI 4115 oxidized specimens at 1000 °C according to exposure time in the furnace: (**a**) 2 min; (**b**) 5 min; (**c**) 10 min; (**d**) 20 min; (**e**) 50 min; (**f**) 100 min.

Figure 4 contains microstructure images of the AISI 4115 steel observed under an optical microscope, showing the cross-section of the oxidized specimen according to the oxidation time at 1000 °C. The oxidized layer and the parent material unaffected by oxidation can be clearly distinguished in the cross-sectional photographs of the oxidized specimen,. The dark gray part on the surface corresponds to an oxide layer made of iron oxide formed by high temperature. It is difficult to clearly distinguish the types of iron oxide in the optical photographs; however, it is possible to deal with them through phase analysis, as described next. Overall, the longer the oxidation time, the thicker the oxide layer tends to be, which can essentially be considered as due to the inward diffusion distance of oxygen and outward diffusion of iron in the direction of the base material becoming longer in proportion to the time in a high-temperature oxidation environment.

**Figure 5:**
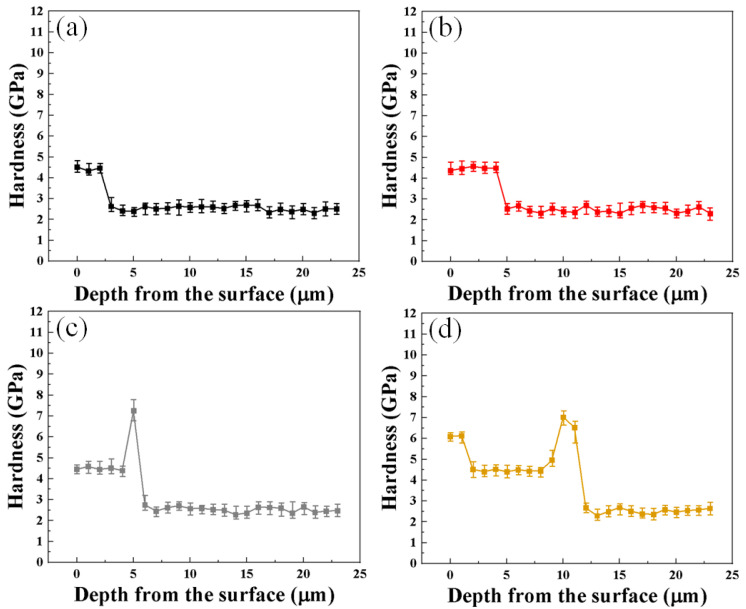
Hardness depth profiles of oxidized AISI 4115 according to oxidation time at 1000 °C: (**a**) 2 min; (**b**) 10 min; (**c**) 20 min; (**d**) 100 min.

Figure 5 shows the hardness profile results from the surface of the AISI 4115 oxidized specimen according to oxidation time. Overall, it was confirmed that the thickness of each detailed oxide layer deepened as the oxidation time increased. The details of the oxide layer composition show different hardness behaviors depending on the position. The hematite, magnetite, and chromite phases have rhombohedral, octahedral, and spinel cubic crystal structures, respectively, and the hardness is known to be about 6.2, 4.5, and 8.1 GPa, respectively; thus, these were used to classify the detailed phases by reference to [25,26,27]. In the case of the specimen oxidized for 2 min, the hardness of the outer oxide layer to be measured (hematite) initially did not appear; instead, magnetite with a hardness of 4.2~4.8 GPa was measured with a uniform thickness of about 2 μm. The hardness of the core part had a range of 2.1 to 2.9 GPa, and the hardness of an internal oxide film (chromite) close to that of the core was not observed. On the other hand, in the case of the specimen oxidized for 100 min the hardness of the outer oxide film (hematite), the middle oxide film (magnetite), and the inner oxide film (chromite) were measured as 5.9~6.3 GPa, 4.4~4.9 GPa, and 6.5~7.0 GPa, respectively. The overall thickness of the oxide layer classified through the hardness profile was judged to be similar to the thickness of the oxide layer observed with the optical microscope shown in Figure 4.

**Figure 6:**
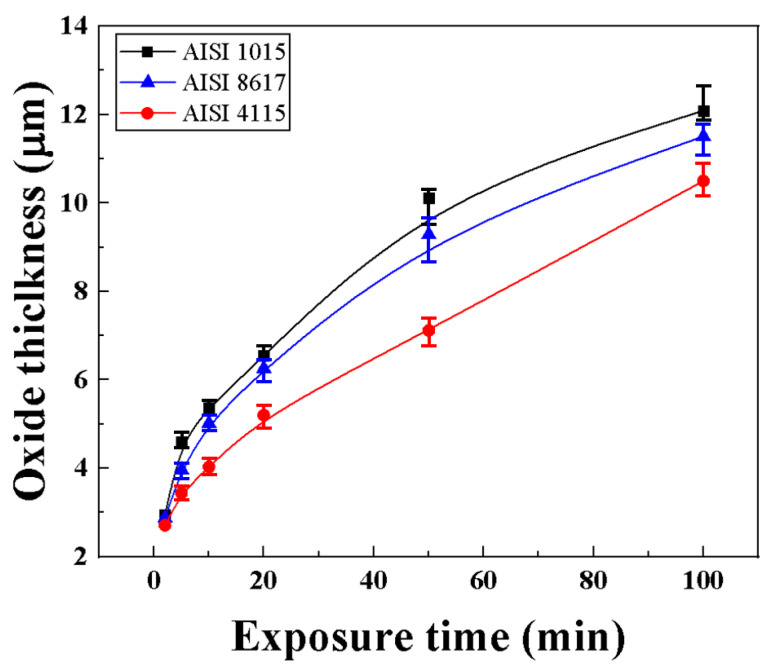
Oxide thickness versus exposure time under air oxidation for various steel types.

Figure 6 shows the thickness of the oxide layer as a graph with reference to the optical photographs of the oxidized specimens according to the oxidation time for each steel type. Regardless of the type of steel, all specimens show a tendency for the thickness to increase rapidly with oxidation time and then gradually decrease. This is because the area of the oxide film formed by the diffusion of oxygen in the initial stage of oxidation increases with time, thereby reducing the inward diffusion of oxygen. Although there is no significant difference in the thickness of the oxide layer depending on the steel type in the initial stage of oxidation, in the steel types containing Cr (AISI 8617, AISI 4115) FeCr_2_O_4_ is formed at the interface between the oxide film and the base material, slowing down the oxidation rate. The longer the oxidation time, the greater the difference in the thickness of the oxide layer by steel type.

**Figure 7:**
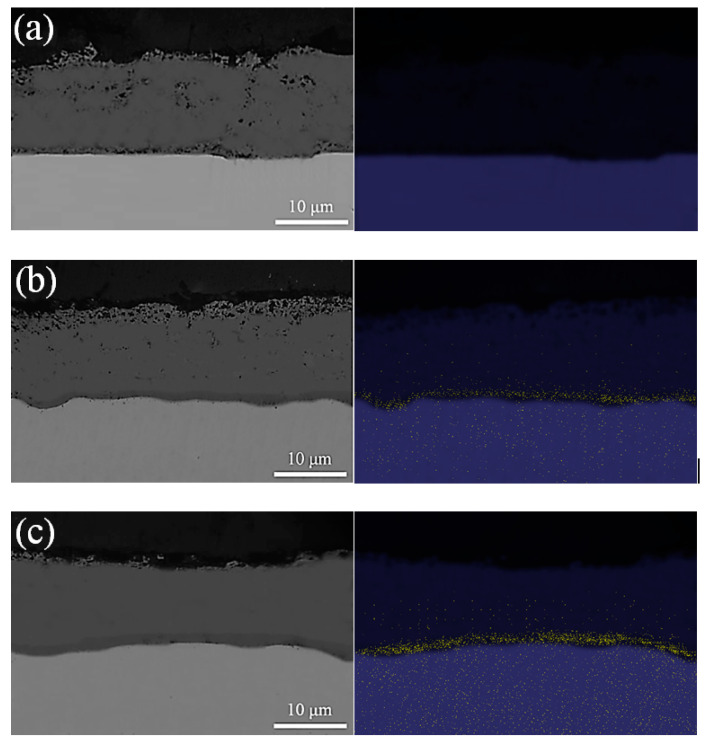
Cross-sectional morphology and EDS elemental mapping analysis according to steel type: (**a**) AISI 1015; (**b**) AISI 8617; (**c**) AISI 4115 steel.

Figure 7 shows the cross-sectional SEM structure of the oxidized specimen at 1000 °C for 100 min at high temperature according to the steel type, along with a picture of the Cr mapping obtained through EDS. From the SEM micrographs, AISI 1015, AISI 8617, and AISI 4115 have respective oxidation thicknesses of 12.14, 11.51, and 10.42 μm. In the optical photographs, it is difficult to distinguish the chromite structure; however, it is judged to be an inner oxide composed of Cr because the black part near the base material of the steel type containing Cr matches the Cr-rich part from the result of mapping the Cr element. In AISI 8617 and AISI 4115, the Cr-rich region was similarly observed at about 4 μm, with the density of Cr distribution being remarkably high in 4115. On the other hand, no corresponding black layer was observed in the AISI 1015 steel that did not contain Cr.

**Figure 8:**
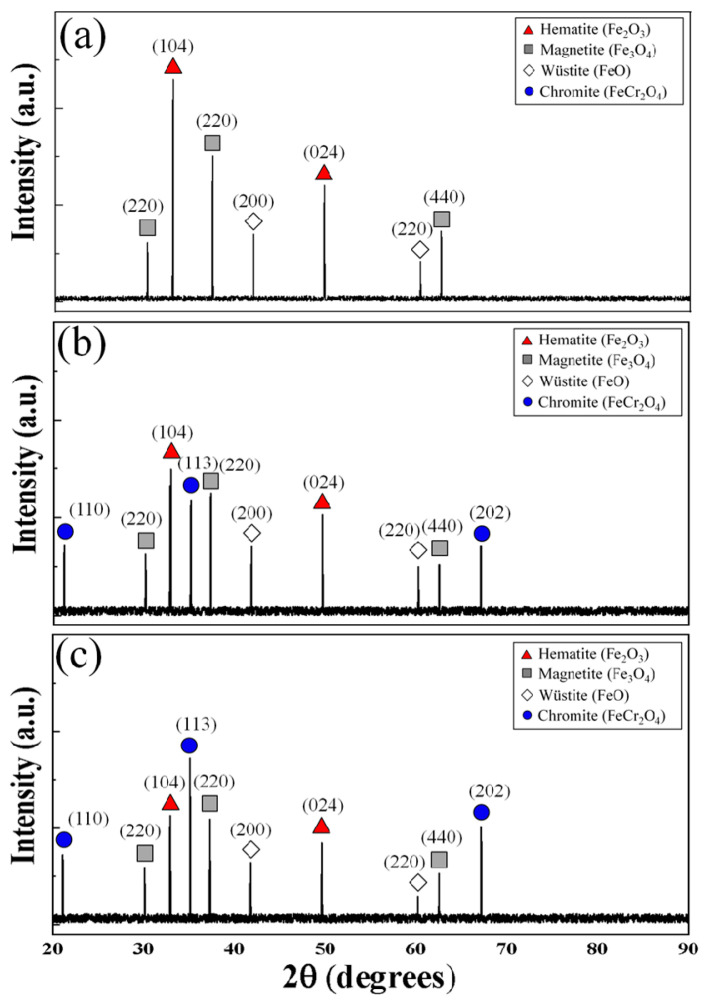
XRD data for the oxide scales obtained according to steel types: (**a**) AISI 1015; (**b**) AISI 8617; and (**c**) AISI 4115 at 1000 °C.

The results of X-ray diffraction performed on the surfaces of the different steel specimens that were high-temperature oxidized at 1000 °C for 100 min are shown in Figure 8. The analysis depth was set with 15 μm as the depth that could be analyzed in consideration of the oxide layer thickness determined from the cross-sectional tissue observation in Figure 4. Three iron oxide phases (hematite, magnetite, and wüstite) were detected on the surface of the oxide specimen by steel type, and the chromite phase was detected in the AISI 8617 and AISI 4115 steels containing Cr. As the Cr content increases, the peak of the chromite increases and the overall depth of the oxide layer becomes thinner, meaning that the peaks of the three iron oxides tend to decrease. The hematite phase located on the very surface of the oxide layer was detected at 2θ = 32.9, 49.6° with (104), (024) planes. The intermediate magnetite phase was detected with (220), (311), and (440) planes at 2θ = 30.1, 37.3, and 62.5°, respectively, where the largest amount of the detailed phase of the oxide film was detected. Finally, the wüstite phase was detected with (200) and (220) sides at 2θ = 41.8, 60.2°, respectively. In particular, as an inner oxide, the chromite phase was detected at 2θ = 21.1, 35.1, and 67.1° with sides (110), (113), and (202), respectively.

**Figure 9:**
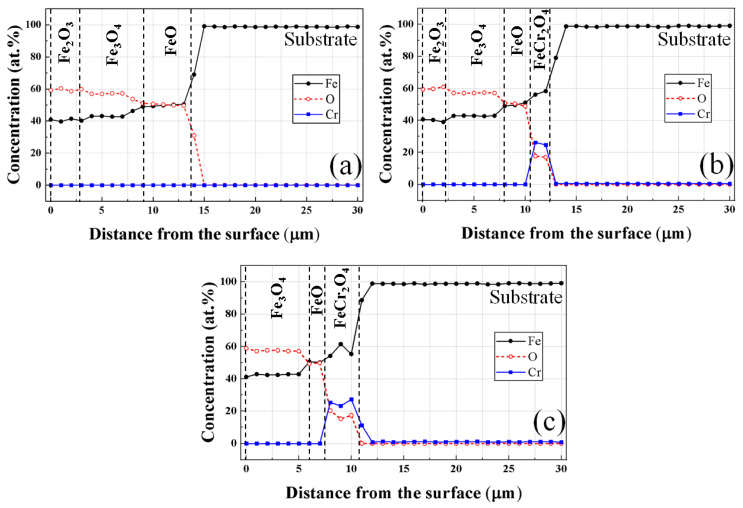
EPMA line profiles for the oxide scale according to steel types: (**a**) AISI 1015; (**b**) AISI 8617; and (**c**) AISI 4115 at 1000 °C.

Figure 9 is the EPMA component profile measured via the line scanning method with the atomic percentage of Fe, O, and Cr atoms used to analyze the components of the oxide layer of the oxidized specimens subjected to high-temperature oxidation at 1000 °C for 100 min. Because there is a clear difference in the composition ratio of the number of atoms constituting the iron oxide depending on the form, it is possible to classify the phases according to the absolute composition ratio of each oxide phase [28,29]. In the case of AISI 1015 and AISI 8617, the composition (Fe:O = 2:3) judged to be hematite phase on the outermost surface was analyzed in the range of 39.1:60.9~41.4:58.6 (at.%). On the other hand, in the AISI 4115 steel grade, although hematite was detected in the X-ray diffraction analysis (Figure 8), it was judged not to be detected in the EPMA component profile result because the outer oxide film is thinner than the electron beam influence range. The magnetite phase (Fe:O = 3:4) and wüstite phase (Fe:O = 1:1) were prominent in all steel grades, ranging from 42.2:57.8~43.5:56.5 and 48.9:51.1~52.2:47.8 (at.%), respectively. In particular, based on the region where Cr elements are distributed in Figure 7b, the region where Cr was detected was judged to be inner oxide (chromite) in agreement with the EPMA analysis [30]. As the Cr content in the steel increases, the overall thickness of the oxide layer becomes thinner and the thickness of chromite oxide increases slightly.

**Figure 10:**
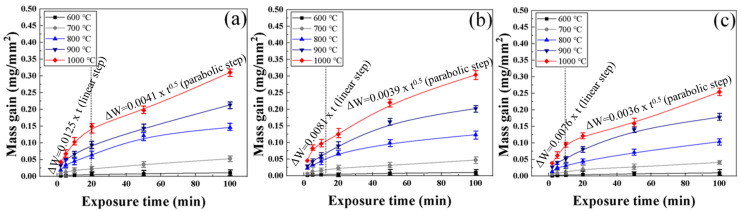
Mass gain curves of high-temperature oxidation according to exposure time: (**a**) AISI 1015; (**b**) AISI 8617; (**c**) AISI 4115.

Figure 10 shows the mass gain curve according to oxidation time for each steel type. As the oxidation temperature and exposure time increase, the mass gain per unit area increases. This is because the diffusivity of oxygen is increased at higher oxidation temperature, meaning that a large amount of oxygen diffuses into the substrate, and the mobility of Fe cations is increases [31,32]. In addition, the amount of oxygen entering becomes sufficient and the diffusion distance increases as the oxidation time increases, and the mass gain consequently increases due to the formation of a thicker oxide film. By comparing Figure 10a–c, it can be noted that the oxidation mass gain curves according to steel type are distinctly different. The rate of mass gain according to the oxidation time decreases in the order AISI 1015 (0 wt.%Cr), AISI 8617 (0.52 wt.%Cr), AISI 4115 steel (0.98 wt.%Cr), and the time of the linear step decreases as well.

Based on the mass gain curve described in Figure 10, the oxidation rate (experimental Kι) was fitted in a linear step according to the oxidation temperature for each steel type, as shown in Table 2. Because the oxidation reaction in the linear step is greatly affected by the oxygen fraction introduced from the outside, the effect of oxidation temperature and Cr content on the linear oxidation rate was investigated with the same flow rate (50 sccm air). For all steel types, the experimental K_ι_ increases as the oxidation temperature increases. For example, in the case of AISI 1015, the oxidation rate in the linear range is increased from 1.73 × 10^−4^ mg·mm^−2^·min^−1^ (600 °C) to 1.25 × 10^−2^ mg·mm^−2^·min^−1^ (1000 °C) an increase of about 72 times. The higher the Cr content, the lower the linear oxidation rate. However, because the Cr content of the steel used in the experiment was less than 1 wt.%, the difference in oxidation rate was actually insignificant.

Experimental K_p_ is shown in Table 3 by fitting the mass gain curve in the section where the oxidation reaction forms a parabolic step. In the linear step, the oxidation reaction increases rapidly with the same slope, whereas in the parabolic step the oxidation reaction completely covers the surface and reaches saturation according to the oxidation time. The experimental K_p_ increases as the oxidation temperature increases for all steel types. In particular, the K_p_ value decreases as the steel type contains more Cr.

According to NevioBalo and Arrhenius [33], the relationship between the parabolic rate constant (*K_p_)* and activation energy can be expressed by Equation (3):

(3) $$K_{p}=K_{0}\cdot exp(-\frac{Q}{RT})$$

where *K*_0_ is the model constant, *Q* is the activation energy of the steel types (J/mol), T is the oxidation temperature (*K*), and *R* is the gas constant (8.314 J/mol·K). Taking the logarithm function on both sides of Equation (4), this can be expressed as follows:

(4) $$\ln K_{p}=\ln K_{0}+(-\frac{Q}{RT})$$

**Figure 11:**
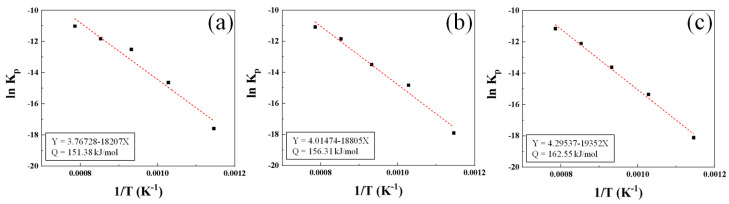
Calculated fitting curve according to steel grade: (**a**) AISI 1015; (**b**) AISI 8617; (**c**) AISI 4115.

By fitting the ln *K_p_* graph according to 1/*T* using the *K_p_*, *R*, and *T* values of the steel types according to the oxidation temperature (Equation (4)), we obtain the results shown in Figure 11; the slope of the line means (−*Q*/*R*). The activation energy of the three steel types used in the experiment are calculated as 151.38 kJ/mol (AISI 1015), 156.31 kJ/mol (AISI 8617), and 162.55 kJ/mol (AISI 4115). Therefore, it is confirmed that with lower Cr content, the activation energy is lower and the oxidation rate is higher, as the barrier to the oxidation reaction is lower.

## 4. Conclusions

In this work, low-alloy steel specimens differing in Cr content were oxidized for a range of oxidation temperatures and times, their oxidation behavior was quantitatively studied through oxidation mass gain and phase analysis, and a model formula was derived according to the oxidation kinetics results.

1. As the oxidation time increased, the thickness of the oxide layer increased as well, and spalling occurred on the outermost surface when oxidized for a long time.
2. The higher the Cr content, the higher the density of the Cr-rich zone inside the wüstite that formed near the parent material; because the Cr ions are able to effectively diffuse outward to form chromite, the transition time from the linear to the parabolic step during the diffusion step is shortened.
3. Because the steel used in this research had a higher Cr content, the overall oxidation rate constants (K_l_, K_p_) decreased and the activation energy change of the oxidation reaction showed a clear difference according to steel type.
4. By deriving the activation energy for the oxidation reaction according to the Cr content, the oxidation behavior can be predicted in various temperature ranges.

## Figures and Tables

**Table 1:** Composition of the tested steels (wt.%).

	C	Cr	Si	P	Mn	S	Fe
AISI 1015	0.159	-	0.21	0.03	0.64	0.029	balance
AISI 8617	0.167	0.52	0.19	0.06	0.73	0.023	balance
AISI 4115	0.163	0.98	0.28	0.02	0.61	0.014	balance

**Table 2:** The calculated oxidation mass gain linear rate constant at different temperatures.

**Temperature (°C)**	**K_ι_ (mg·mm^−2^·min^−1^)**		
	**AISI 1015**	**AISI 8617**	**AISI 4115**
600	1.73 × 10^−3^	1.45 × 10^−4^	1.54 × 10^−4^
700	2.18 × 10^−3^	1.41 × 10^−3^	6.93 × 10^−4^
800	5.23 × 10^−3^	4.06 × 10^−3^	3.12 × 10^−3^
900	5.96 × 10^−3^	4.61 × 10^−3^	4.32 × 10^−3^
1000	1.25 × 10^−2^	8.09 × 10^−3^	7.66 × 10^−3^

**Table 3:** The calculated oxidation mass gain parabolic rate constant at different temperatures.

Temperature (°C)	K_p_ (mg^2^·mm^−4^·s^−1^)		
	AISI 1015	AISI 8617	AISI 4115
600	2.32 × 10^−8^	1.69 × 10^−8^	1.37 × 10^−8^
700	4.47 × 10^−7^	3.66 × 10^−7^	2.17 × 10^−7^
800	3.74 × 10^−6^	1.38 × 10^−6^	1.22 × 10^−6^
900	7.38 × 10^−6^	7.23 × 10^−6^	5.59 × 10^−6^
1000	1.66 × 10^−5^	1.53 × 10^−5^	1.41 × 10^−5^

## Data Availability

Not applicable.
